# 658. Evaluation of Effectiveness of Cefmetazole vs Meropenem for Invasive Urinary Tract Infections Caused by ESBL-Producing *Escherichia coli*: A Prospective Multicenter Observational Study

**DOI:** 10.1093/ofid/ofac492.710

**Published:** 2022-12-15

**Authors:** Kayoko Hayakawa, Kohei Uemura, Yasufumi Matsumura, Aki Sakurai, Ryutaro Tanizaki, Koh Shinohara, Takehiro Hashimoto, Hideaki Kato, Takashi Matono, Ryota Hase, Momoko Mawatari, Hiroshi Hara, Yukihiro Hamada, Sho Saito, Yohei Doi

**Affiliations:** National Center for Global Health and Medicine, Shinjuku-ku, Tokyo, Japan; The University of Tokyo, Bunkyo-ku, Tokyo, Japan; Kyoto University Graduate School of Medicine, Kyoto, Kyoto, Japan; Fujita Health University, Aichi, Aichi, Japan; Ise Municipal General Hospital, Ise-shi, Mie, Japan; Kyoto University Graduate School of Medicine, Kyoto, Kyoto, Japan; Oita University Hospital, Yufu-shi, Oita, Japan; Yokohama City University Hospital, yokohama-shi, Kanagawa, Japan; Iizuka Hospital, Fukuoka, Fukuoka, Japan; Japanese Red Cross Narita Hospital, Narita-shi, Chiba, Japan; Japanese Red Cross Medical Center, Tokyo, Tokyo, Japan; Yokohama Municipal Stroke and Neurospine Center, Kanagawa, Kanagawa, Japan; Tokyo Women's Medical University, Tokyo, Tokyo, Japan; National Center for Global Health and Medicine, Shinjuku-ku, Tokyo, Japan; Fujita Health University, Aichi, Aichi, Japan

## Abstract

**Background:**

ESBL-producing *E. coli* (ESBLEC) continues to increase worldwide. For the infection due to ESBLEC, no antimicrobial agent has clearly demonstrated therapeutic effectiveness comparable to that of carbapenem. Overuse of carbapenems may lead to an increase in carbapenem-resistant bacteria. Cefmetazole (CMZ) is active against ESBLEC, however, there are limited multicenter studies on the effectiveness of CMZ, an important carbapenem-sparing therapy for the treatment of ESBLEC.

**Methods:**

This prospective, observational study included patients hospitalized for invasive urinary tract infection (iUTI) due to ESBLEC between March 2020 and November 2021 at 10 centers in Japan, with either CMZ or meropenem (MEM) initiated within 96 hours of culture submission as definitive therapy, and used for at least 4 days. The diagnosis of iUTI was made in patients with a fever of ≥37.5°C, symptom of pyelonephritis such as back pain, pyuria, and ESBLEC detected in urine (≥10^4 CFU/mL). Outcomes included clinical effectiveness (resolution of all clinical symptoms or improvement to pre-infection status) between day 4 to 6 of treatment (early) and between the final day of treatment and 2 days later (late), microbiological effectiveness (reduction to ≤10^3 CFU/ml) between day 4 to 6, and mortality. Outcomes were adjusted for the inverse probability of propensity scores (PS) for receiving CMZ or MEM treatment.

**Results:**

Seventy-seven patients in the CMZ group and 46 in the MEM group were included. In univariate analysis, the CMZ group was older than the MEM group, although the MEM group had higher qSOFA, CRP, more frequent medical device use and concurrent bacteremia at the start of study drugs than the CMZ group (Table). Univariate analysis showed no difference in clinical effectiveness, and 30-day mortality was higher in the MEM group. In all cases with available data (CMZ: n=57, MEM: n=22), both drugs were microbiologically effective. After PS adjustment, clinical effectiveness did not differ between the two groups. The risk of 30-day mortality was lower in CMZ group, whereas the risk of recurrence was similar in both groups.

**Figure:**
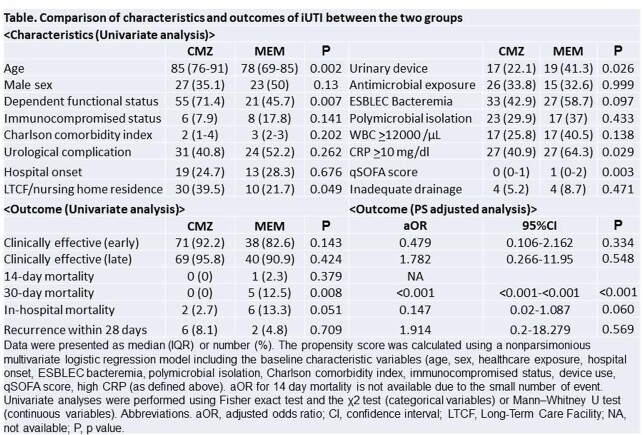

**Conclusion:**

CMZ is as effective as MEM for the treatment of iUTI, suggesting that it is a promising carbapenem-sparing therapy.

**Disclosures:**

**Sho Saito, MD, PhD**, DAIICHI SANKYO: Grant/Research Support|EVEC: Grant/Research Support|EVEC: Patents obtained through collaborative research|SHIONOGI: Grant/Research Support|Takeda: Advisor/Consultant.

